# Localizing Isomerized Residue Sites in Peptides with Tandem Mass Spectrometry

**DOI:** 10.1021/jasms.3c00373

**Published:** 2024-03-05

**Authors:** Hoi-Ting Wu, Brielle L. Van Orman, Ryan R. Julian

**Affiliations:** Department of Chemistry, University of California, Riverside, California 92521, United States

**Keywords:** Fragmentation, Isomer, Epimer, isoAsp, Leucine, Isoleucine

## Abstract

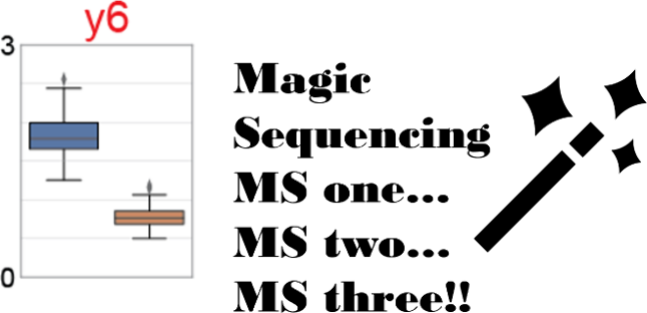

Isomerized amino acid residues have been identified in many peptides extracted from tissues or excretions of humans and animals. These isomerized residues can play key roles by affecting biological activity or by exerting an influence on the process of aging. Isomerization occurs spontaneously and does not result in a mass shift. Thus, identifying and localizing isomerized residues in biological samples is challenging. Herein, we introduce a fast and efficient method using tandem mass spectrometry (MS) to locate isomerized residues in peptides. Although MS^2^ spectra are useful for identifying peptides that contain an isomerized residue, they cannot reliably localize isomerization sites. We show that this limitation can be overcome by utilizing MS^3^ experiments to further evaluate each fragment ion from the MS^2^ stage. Comparison at the MS^3^ level, utilizing statistical analyses, reveals which MS^2^ fragments differ between samples and, therefore, must contain the isomerized sites. The approach is similar to previous work relying on ion mobility to discriminate MS^2^ product ions by collision cross-section. The MS^3^ approach can be implemented using either ion-trap or beam-type collisional activation and is compatible with the quantification of isomer mixtures when coupled to a calibration curve. The method can also be implemented in combination with liquid chromatography in a targeted approach. Enabling the identification and localization of isomerized residues in peptides with an MS-only methodology will expand accessibility to this important information.

## Introduction

**Scheme 1:**
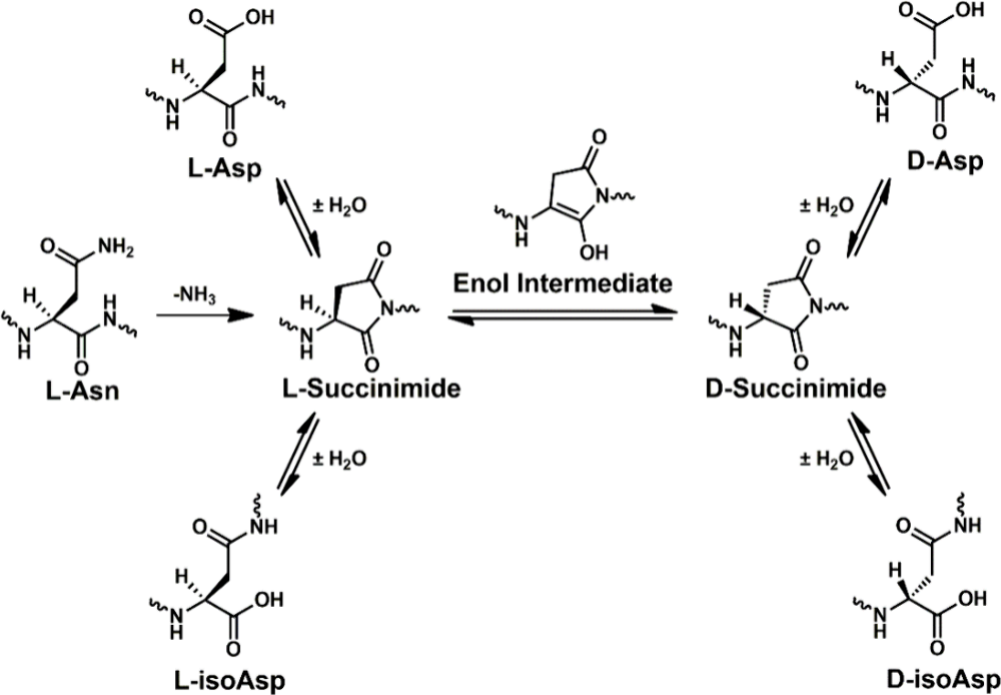
Isomerization Mechanism for Aspartic Acid (Asp) Involves the Succinimide Ring Formation

Isomerization of amino acids alters protein structure and biochemical properties. Purpose-built d-amino acid-containing peptides (DAACP) have been found in plants and animals,^1−7^ while isomerized residues resulting from spontaneous chemistry have also been identified in long-lived proteins (LLPs) extracted from aged tissue samples in humans.^8^ Recent work has shown striking relationships between isomerization and age-related diseases such as Alzheimer’s.^9^ Spontaneous isomerization is also an important consideration in the preparation, characterization, and storage of protein-based pharmaceuticals.^10^ Aspartic acid (Asp) and serine (Ser) are the most susceptible to isomerization in LLPs. Over extended timeframes, Asp forms a succinimide ring that reopens through hydrolysis to yield four isomers: l-Asp, d-Asp, l-isoAsp, and d-isoAsp (Scheme 1).^11^ A methylene group is inserted into the protein backbone with the formation of either isoAsp, which forms a kink in the backbone and can alter tertiary protein structure.^12,13^ Isomerization can have profound effects on protein structure and biochemical properties, which makes identifying isomerized residues important for enhancing our understanding of age-related pathology.^14^

Despite having the same mass, peptide isomers can be differentiated by using mass spectrometry based on their fragmentation patterns. Peptide isomers by definition have different structures, which may lead to differences in fragmentation.^15^ Radical-directed dissociation (RDD) utilizes the migration of a radical to generate fragments, yielding patterns sensitive to structure that can differentiate peptide isomers.^16,17^ Recently, our group introduced a statistical framework that allows the differentiation of isomers with several fragmentation methods including collision-induced dissociation (CID) and higher-energy collisional dissociation (HCD).^18^ This framework provides a more accessible pathway to isomer identification, but localization of the isomerized site is not revealed by differences in the fragment-ion abundance at the MS^2^-level.

Existing analytical methods for mapping isomerization sites in peptides have made significant progress. Liquid chromatography (LC) is the most common technique used to separate and confirm the presence of peptide isomers based on their fragmentation patterns.^19,20^ The isomerized residue can be identified by matching the retention time to that of a synthetic peptide with a known isomerized residue. This process can be time-consuming and expensive if many standards must be synthesized, which can easily occur for uncommon or multiple-modification sites. In addition to LC, ion mobility spectrometry mass spectrometry (IMS-MS) can be used to separate peptide isomers.^21,22^ When IMS is performed on fragment ions, differences in arrival time distributions can be used to localize the isomerized residues.^23−25^ While IMS offers faster separation (in milliseconds) than LC (in minutes), it is not as widely available. Another method for identifying isomerized residues utilizes an enzymatic treatment. Due to their inherently different structures, isomerized residues either enable or prevent the action of enzymes.^26−28^ For example, isomerized Asp residues are resistant to digestion by proteases, which can be leveraged in several ways for identification.^29^ If an Asp-specific protease such as Asp-N is employed, information about localization can also be inferred.^30^ The repair enzyme protein l-isoaspartyl methyltransferase (PIMT), which specifically targets l-isoAsp, can also be used for localization and identification.^31,32^

Herein, we propose a simple method to locate isomerized residues using tandem mass spectrometry to reveal differences in the fragmentation of fragment ions (MS^3^ experiments). We demonstrate successful differentiation of the most common and most difficult isomerized residues, including all four Asp isomers and epimers of Ser, alanine (Ala), proline (Pro), and leucine (Leu)/isoleucine (Ile). The method allows reliable localization of isomerized sites without the use of IMS or the synthesis of a large number of peptide standards.

## Method

### Materials

Organic solvents and reagents were purchased from Fisher Scientific and Sigma-Aldrich and were used without further purification. Fmoc-protected amino acids and Wang resins were purchased from Anaspec Inc. or Chem-Impex International.

### Peptide Synthesis

Peptides were manually synthesized following an accelerated FMOC-protected solid-phase peptide synthesis protocol with Wang resin employed as the solid support.^33^ Following synthesis, peptides were stored frozen at −20 °C in 50/50 acetonitrile/water (v/v).

### Mass Spectrometry Analysis

All peptides were prepared in Optima water + 0.1% formic acid with a final concentration of 5 μM. Peptides were analyzed on a Thermo Fisher Scientific Orbitrap Fusion Lumos Tribrid mass spectrometer using static nanoESI infusion. The capillary temperature, RF voltage, resolution, and spray voltage were set to 275 °C, 50%, 30k, and 1.2–1.5 kV, respectively. Each series of peptide isomers were examined under the same MS parameters and fragmented by the same fragmentation energy and isolation window width. To ensure the quality of the MS^3^ spectra, such as the signal-to-noise ratio, an ion count cutoff of 5.0 × 10^6^ was set for the MS^2^ fragments. The AGC and maximum injection times were set to 1000% and 100 ms for MS^3^ experiments. Each b/y ion was fragmented again in MS^3^ by CID or HCD. After stabilizing the spray with a relative standard deviation (RSD) < 15% for the total ion count, 100 scans were collected for all experiments.

### Liquid Chromatography–Mass Spectrometry Analysis

LC-MS data was acquired using a Thermo Fisher Ultimate 3000 RSLCnano system coupled to an Orbitrap Fusion Lumos. Approximately 1 ng of a 2:1 mixture of synthetic l-Asp and d-isoAsp standards of the FAEDVGSNK peptide were injected and separated on a column packed with ReproSil-Pur 120 C18-AQ 1.9 μm beads (Dr. Maisch). The gradient was 0.875% B per minute where buffer A was Optima water with 0.1% formic acid and buffer B was 80/20 acetonitrile/Optima water with 0.1% formic acid. Flow rate was 0.3 μL/min. The MS method used a tSIM scan of the doubly charged precursor followed by data dependent CID MS^2^ on the [M + 2H]^2+^ peak in the ion trap, with one scan acquired per cycle. A targeted mass filter was used to select the b5^+^ and y6^+^ fragment ions for CID MS^3^ scans in the ion trap. Ten MS^3^ scans of each fragment ion were acquired per cycle by using rapid scan speed.

### Data and Statistical Analyses

The ion counts of the most intense ions were extracted in each scan. The *R*-value program (available to download at https://sites.google.com/ucr.edu/jlab) was used to identify the MS^3^ fragments with the highest dissimilarity in relative intensity between spectra. The mass tolerance and peak threshold were set to 0.01 Da and 10% relative intensity. Higher variation is observed in ^13^C isotopes; thus, they were excluded from intensity ratio calculations.^17^ The intensity ratios of the fragment ions identified by the *R* value were then calculated for each individual scan. A two-sample *t*-test was used to determine if the means from two experiments are statistically different. Statistical power reports the probability of a significance test detecting an effect when there truly is one (on a scale of 0–1). The effect size measures the magnitude of the differences between samples. An effect size of 0.8 is typically considered large in biological and social studies.^34^ Statistical analyses of two-sample *t*-tests and effect size (η^2^) were performed in Excel on the intensity ratios. Statistical power (1 – β) calculations were performed by using Statsmodels 14.0 in Python 3.11. Assuming the number of samples (or scans) is the same for isomer A and isomer B, the effect size is calculated using
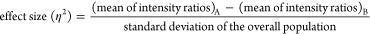


## Results and Discussion

As mentioned above, structural differences in peptide isomers lead to differences in MS^2^ fragmentation patterns that can be utilized for isomer identification. Frequently, these differences can only be detected in terms of the fragment ion intensities, which vary when the isomerized residues influence the energetics of the transition states leading to dissociation. Furthermore, although useful for identification, MS^2^ data cannot reliably locate the specific residues that are isomerized because isomerization can influence fragmentation at sites that are remote in sequence but proximal when considering the folded structure. However, ion mobility experiments have demonstrated that structural differences are also detectable in CID fragments containing isomerized sites, which can be used to localize sites of isomerization based on differences in collision cross-section.^23,24^ Based on these previous observations, we hypothesized that fragments containing isomerized residues might also be identifiable by differences in fragmentation patterns at the MS^3^ level, enabling isomer site localization by tandem MS without ion mobility. The origin of differences at the MS^3^ level would again result from variations in how the isomerized sites would impact the transition states, leading to dissociation.

### Workflow and Data Processing

**Figure 1:**
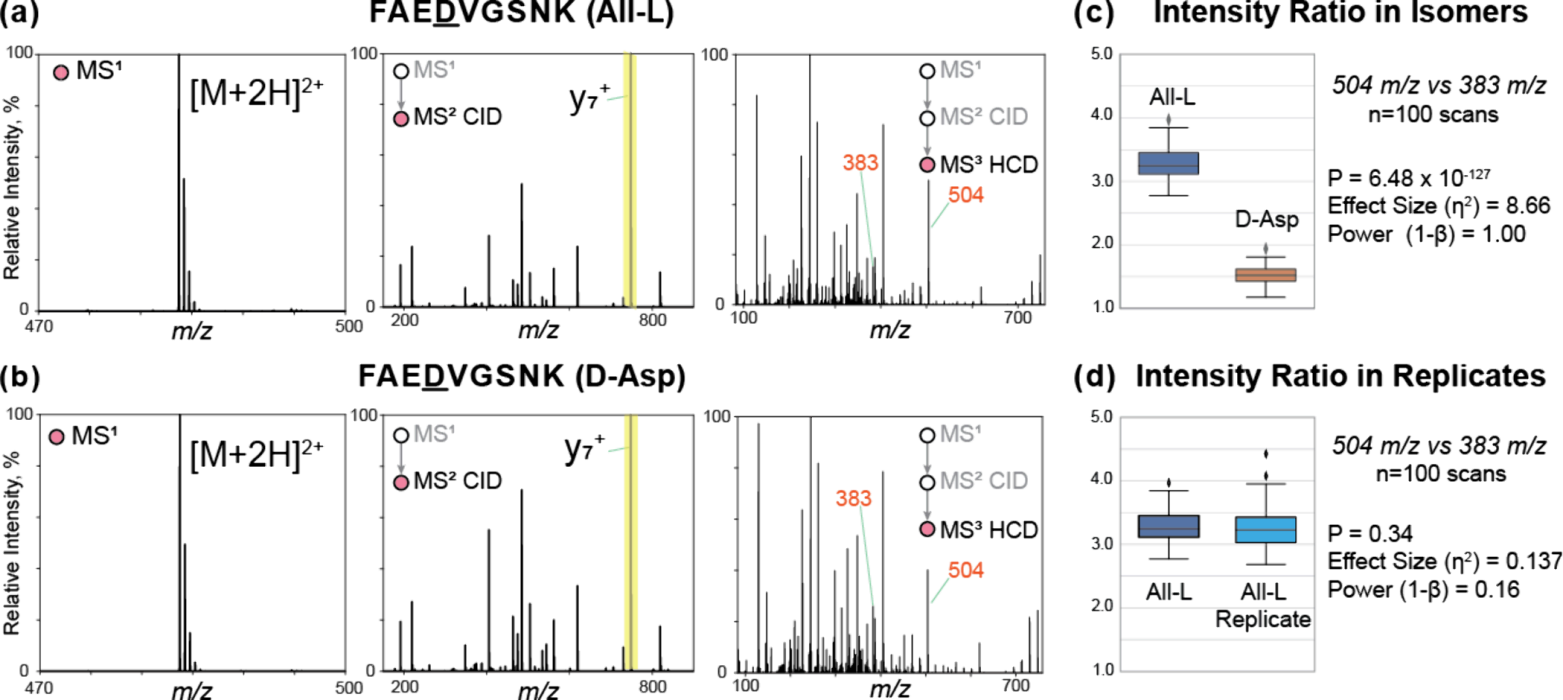
MS spectra at annotated levels for FAEDVGSNK [M + 2H]^2+^: (a) All-l and (b) d-Asp. MS^1^ spectra are identical, as expected. CID fragmentation reveals patterns that differ between the two isomers. Subsequent HCD fragmentation of the y_7_^+^ ions (highlighted in yellow) again yields different fragmentation patterns in MS^3^ spectra. (c) Boxplots of the intensity ratios for All-l (blue) and d-Asp (orange) peptide isomers differ considerably. (d) Boxplots for replicate data are far more similar.

Representative spectra and extracted data are illustrated in Figure 1 for isomers of FAEDVGSNK, a peptide from β-amyloid (residues 20–28) where Asp23 is known to be isomerized.^35^ As expected, the MS^1^ spectra are identical because isomers have the same exact mass. The CID MS^2^ spectra are also very similar and contain all of the same fragment ions, although the intensity of certain ions varies between isomers. In a suitable ion trap instrument, the major fragment ions can be reisolated and fragmented in MS^3^ experiments. In Figure 1, MS^3^ data are shown for fragmentation of the y_7_ ion (highlighted in yellow in the MS^2^ spectra). The MS^3^ spectra are again very similar apart from differences in the intensity of certain ions. MS^3^ data can be similarly acquired for all of the major peaks in the MS^2^ spectrum. To identify MS^3^ spectra that are sufficiently different to be likely to contain isomerized sites, we initially utilized our previous comparison method for identifying differences in MS^2^ spectra that relies on evaluation of the fractional abundance of all fragment ions.^18^ This approach will often work (for example, it correctly distinguishes the MS^3^ spectra in Figure 1), but in other cases the approach will fail. For example, if the MS^3^ spectra contain few peaks, then the statistical confidence that minor differences are meaningful is weakened (Supporting Information Figure S1). Given that many MS^2^ product ions contain only a few residues, many MS^3^ spectra are expected to produce few fragment ions. Additionally, the reproducibility of MS^3^ spectra can vary significantly due to differences in the abundance of the precursor ions. For example, MS^3^ spectra obtained from intense MS^2^ ions will be much more reproducible than those obtained from low-abundance MS^2^ ions and, therefore, easier to distinguish with statistical certainty.

To address this shortcoming, we revisited the *R*-value approach^16^ but sought to include statistical certainty as well. First, the peak ratio that changes the most between MS^3^ spectra of the two isomers is identified. This ratio is then calculated from each individual scan for the isomer data sets. The boxplots in Figure 1c,d illustrate the results for comparison of MS^3^ product ion ratios derived from peaks at 504 *m*/*z* and 383 *m*/*z*. It is clear that the ratio of these ions differs well outside experimental variation when isomer data are compared (Figure 1c), while replicate data yield similar ratios in Figure 1d (as would be expected). In addition, statistical comparators, such as *p*-value, effect size, and power, all confirm that the ratios from isomer data are unlikely to be the result of random chance.

**Scheme 2:**
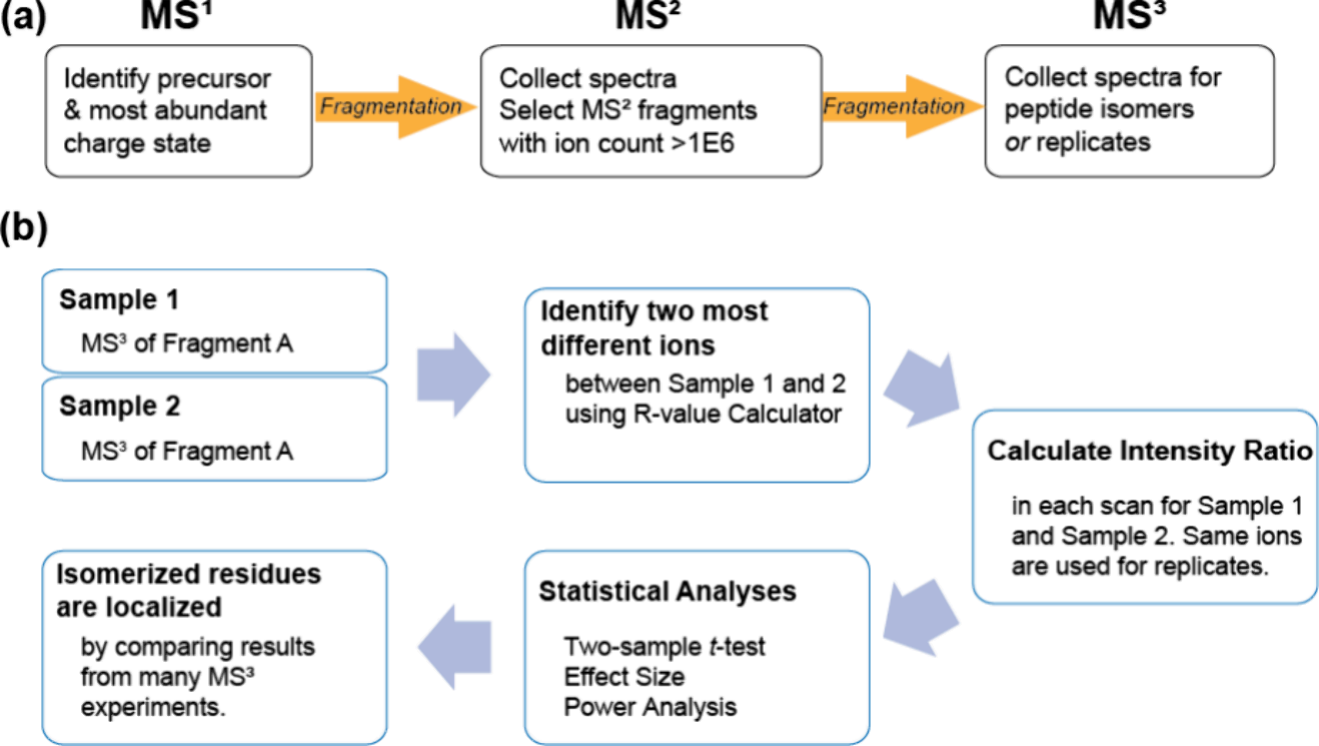
(a) Experimental Workflow for Localization of Isomerized Residue Using Tandem MS and (b) Data Analysis Process for Localization of Isomerized Residue Using MS^3^ Mass Spectra

The overall workflow that will be applied for isomer localization is recapitulated in Scheme 2. The most abundant charge state is identified in MS^1^ and subjected to MS^2^ fragmentation. Ions meeting a threshold ion count were then subjected to MS^3^ as shown in Scheme 2a. For data processing (Scheme 2b), the fragment ion ratio that varies most between isomer MS^3^ data sets will be calculated for each scan and subjected to statistical analysis. Ratios that vary outside typical instrument reproducibility (as determined by the examination of many known samples) will identify fragment ions containing sites of isomerization. By mapping fragments that appear to contain isomerized residues onto the peptide sequence, localization is revealed.

### Locating Isomerized Residue with MS^3^ Fragments

**Figure 2:**
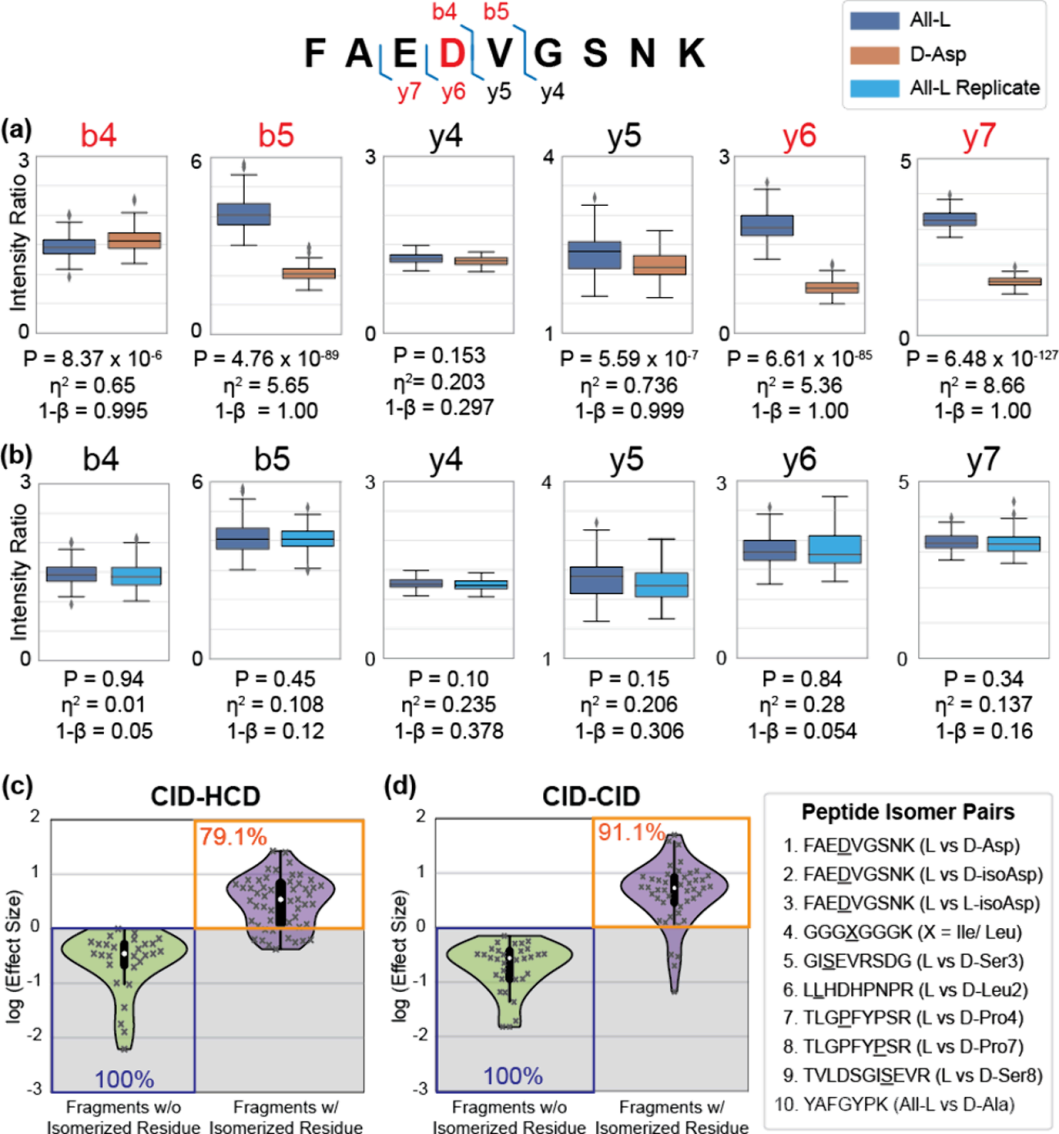
[FAEDVGSNK + 2H]^2+^ precursor produces several CID fragment ions which were fragmented by HCD in MS^3^. The fragment ions containing the isomerized residues are labeled in red. (a) The intensity ratios of the most different MS^3^ fragments between peptide isomers All-l and d-Asp are plotted for each fragment ion along with their corresponding statistical analyses. (b) No statistically significant differences were observed in All-l replicates. Collective results for 10 peptides using CID in MS^2^ followed by either (c) HCD or (d) CID in MS^3^. The log values (effect sizes) of the intensity ratios from two peptide isomers are categorized into fragments with or without the presence of isomerized residues (colored in green and purple). All fragment ions without the isomerized residue have a log_10_ effect size of <0, while most isomeric fragments have a log_10_ effect size of >0.

Detailed results for the canonical and d-Asp isomers of FAEDVGSNK peptide are show in Figure 2a. The CID fragments containing isomerized residues are labeled in red; fragments without modification are labeled in black. The results for the identical analysis of replicate data are shown in Figure 2b. The replicate data yield ratios that are very similar in all cases, with high *p*-values and low effects sizes and low power. Some fragments in the isomer data set yield clearly different ratios (b_5_, y_6_, y_7_), while others do not (b_4_, y_4_, y_5_). In particular, the b_4_ and y_5_ results warrant further discussion. Both fragments yield comparable statistical outputs with low *p*-values, modest effect sizes, and high power. Importantly, the b_4_ fragment contains the isomerized site while the y_5_ ion does not. The b_4_ results can be interpreted as a case where the MS^3^ data are not particularly sensitive to the isomerized residue. This is to be expected, as isomers will not always strongly affect product ion distributions. However, for the y_5_ ion, there should be no difference (in theory) in the MS^3^ data as the isomerized residue is not present. We note that y_5_ corresponds to cleavage at the site of isomerization and is also the complement of the b_4_ ion, introducing the possibility that fragmentation may produce two forms of y_5_ and that the population of these forms may be affected by the isomerized site, which in turn leads to differences in fragment ion abundances. Other possibilities may also account for these results, but more importantly, such variations must be taken into account for confident isomer localization.

To establish cutoffs that can be confidently utilized for isomer localization, we examined data for a series of 10 peptides with known sites of isomerization. Among all statistical parameters, the effect size proves to be the most useful and can be used independently to distinguish all fragments that do not contain the isomerized residue, as shown in the green violin plots in Figure 2c,d for MS^3^ by HCD and CID, respectively. In contrast, a greater overlap is observed if *p*-value or power alone is considered. We note that many of the nonisomer fragment comparisons that yielded low *p*-values were from fragments adjacent to or within two residues of the isomerized sites (similar to the y_5_ ion discussed above). Thus, secondary structural effects may influence p-values, but the effect size differences in these cases are modest, allowing them to be properly assigned. It is convenient to plot the log_10_ of the effect size, which provides a cutoff of zero for isomer identification. The log_10_ of the effect sizes obtained from fragments containing the isomerized sites are shown in the purple plots in Figure 2c,d. Although there is some overlap with the nonisomer data (as quantified by the nonoverlapping percentages in red), the majority of fragments containing isomerized residues yield effect sizes greater than 1 (or greater than zero for log_10_ of the effect size). The overlapping ions likely result from cases where the isomerized site fails to significantly influence fragmentation in the MS^3^ data. Based on these results, we will use a cutoff of zero for identifying fragments containing isomerized residues with the log_10_ of the effect size (hereafter simply referred to as “effect size”).

### Locating Isomers with CID-HCD

**Figure 3:**
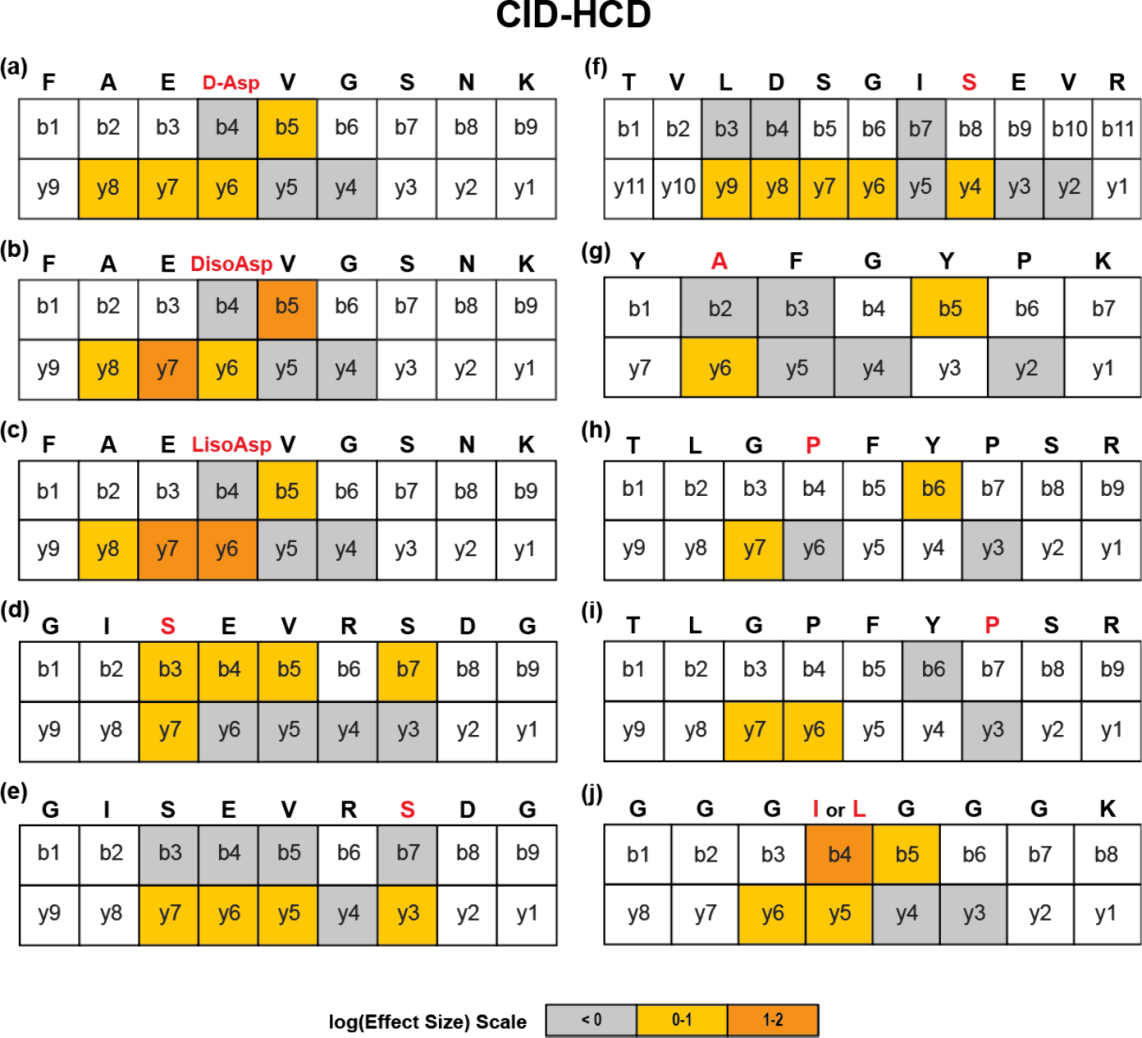
Ten peptide isomers (a–j), where modified amino acids are highlighted in red, were fragmented using CID and followed by HCD to localize sites of isomerization. White boxes indicate ions for which MS^3^ data could not be collected. For all other ions, the color derived from the effect size indicates the probability that a fragment contains an isomerized site (values of >0 are consistent with isomerized sites).

Figure 3 shows the results for mapping effect sizes onto the sequences for a series of 10 peptide isomers. The isomerized residue is indicated in red, and shaded fragments indicate those with sufficient ion count for examination, with the color signifying the magnitude of the effect size. Gray boxes indicate data were collected but did not confirm isomerization, while colored boxes identify isomerized fragments and provide the relative effect size. For example, Figure 3a summarizes the more detailed results shown previously for FAEDVGSNK in Figure 2a. The localization of the isomerized site can then be identified by combining the leftmost colored b ion with the rightmost colored y-ion. For FAEDVGSNK, the isomer is localized within DV. In the context of human biology, this would strongly indicate Asp as the likely isomer, given the absence of any known isomerization at Val. Similar results were obtained for the d-isoAsp and l-isoAsp isomers of FAEDVGSNK.

Epimerization of serine is also known to occur in human long-lived proteins associated with aging.^8,36,37^ GISEVERSDG contains two Ser residues (Ser3 and Ser8) that can be modified. MS^3^ by HCD localizes the Ser3 isomer precisely, but the failure of the b_7_ ion to localize the Ser8 site precludes the precise identification of that isomeric form. TVLDSGISEVR also contains two Ser residues. For this peptide, fragments y_4_ and y_6_ are consistent with isomerization only at Ser8. Although the absence of isomer-positive b ions fails to explicitly narrow the isomerization site beyond the final four residues (SEVR), the y_3_ and y_2_ ions suggest that Ser8 is the most likely site of isomerization (i.e., these ions are unlikely to contain isomerized Glu9 or Val10). These examples demonstrate that our approach is sensitive to Ser epimers and capable of significantly narrowing down the modification site.

Epimerization of Ala, which is the smallest amino acid with a chiral center, is often observed in animals.^38^ For example, d-Ala2 in dermorphin (YAFGYPK) from the skin of *Phyllomedusa* species was identified in 1990.^39^ MS^3^ fragmentation of YAFGYPK by HCD reveals that b_5_ and y_6_ contain isomerized residues and definitively localize the isomerization site to AFGY.

Although proline cis–trans isomerization is more common in proteins and peptides,^40,41^d-proline has been identified in neuropeptides in cicadas.^42,43^ Since the proline effect favors fragmentation of the peptide bond N-terminal to Pro,^44,45^ we investigated whether the proline effect would influence isomer detection of isomerized proline. TLGPFYPSR which derives from human αA-Crystallin (residues 13–21) contains Pro at positions 4 and 7. Due to the proline effect, this peptide does not produce many fragments suitable for MS^3^ analysis (Supporting Information Figure 2). For isomerization at Pro4, the b_6_ and y_7_ ions localize the isomerized residue within GPFY. However, for the Pro7 isomer, the isomerization site can be excluded from only the first two N-terminal residues. These results suggest that highly favored dissociation pathways may hinder isomer localization by limiting the number of available fragments.

It is very difficult to differentiate between the constitutional isomers leucine and isoleucine using collisional activation because the side chains do not fragment, as they do with radical fragmentation methods.^46−48^ It has been demonstrated previously that tandem MS experiments (up to MS^5^ may be required) can identify Leu/Ile by way of a diagnostic 69 Da ion produced by dissociation of the immonium ion of Ile.^49^ GGGXGGGK (X = Leu, Ile) peptide isomers were examined with our MS^3^ method, and the results are shown in Figure 3j. This peptide sequence represents a challenging target because the surrounding glycine residues lack any side chains or chirality that might facilitate structural differentiation. Despite this challenge, Leu/Ile isomers can be precisely localized by using differences in MS^3^ fragmentation patterns.

### Mapping Isomerized Residues with CID-CID

**Figure 4:**
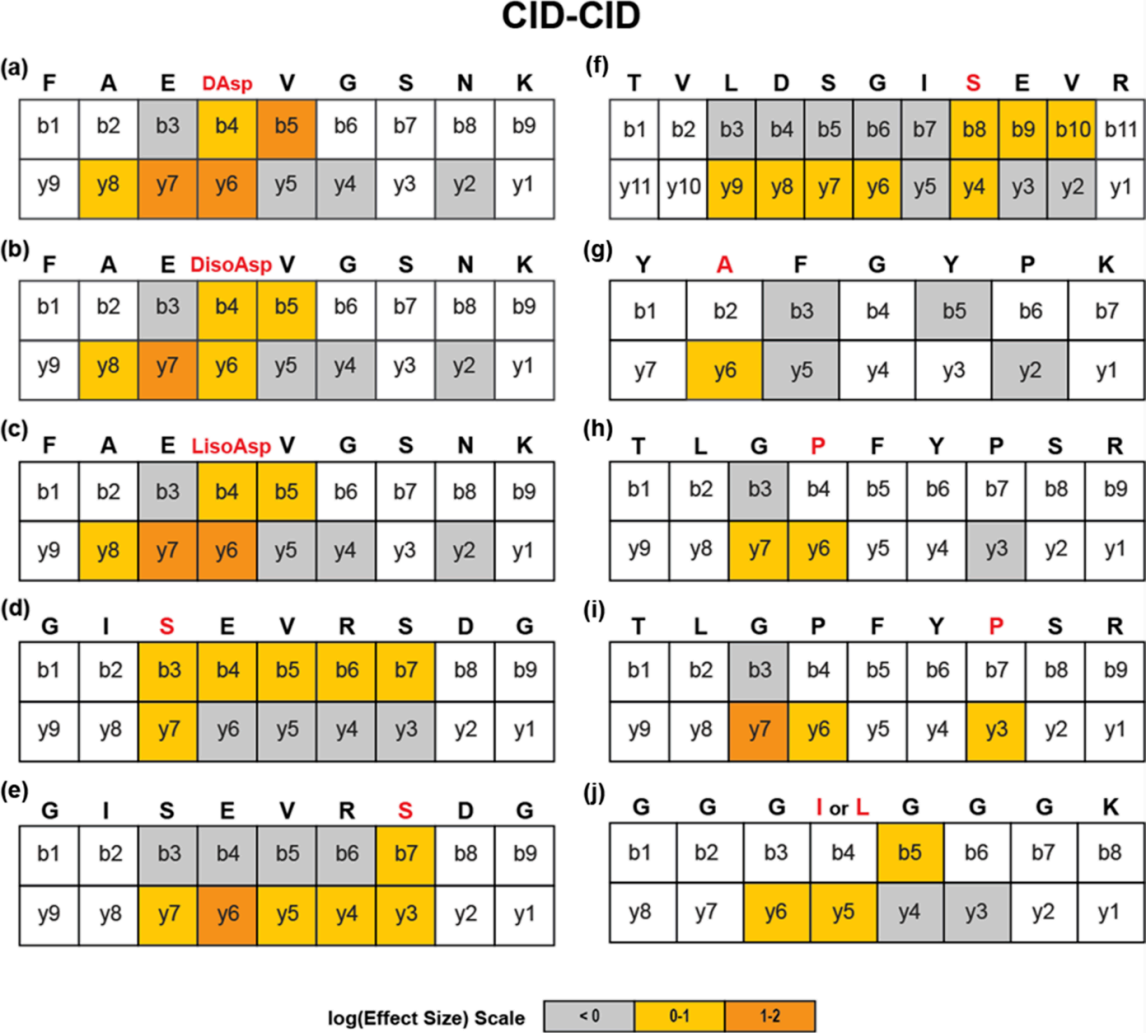
The same peptide isomers (a–j) were fragmented using CID-CID for isomer mapping, where modified amino acids are highlighted in red. The intensity ratio changed due to the isomerized residue is indicated by color as seen in the effect size scale.

A critical parameter of these experiments is the preservation of the ion count at the MS^2^ stage to allow for sufficient ions to be analyzed in MS^3^. CID is therefore preferable over HCD for MS^2^ because fewer fragments are likely to be generated (albeit with reduced sequence coverage).^50^ To evaluate the performance of CID for MS^3^ analysis, we repeated the evaluation of the same peptides from Figure 3 using CID-CID for activation (see Figure 4). For some peptides, marginally better localization is achieved (see Figure 4a–c,e,f), while for others localization is slightly worse (Figure 4g,h). These results suggest that if feasible, both HCD and CID should be utilized for MS^3^ activation to maximize the probability of localizing isomerization sites. We also note that for both methods, y-ions tend to outnumber b-ions that localize isomerization sites, but this may be a result of analyzing tryptic peptides that are more likely to yield y-ions. It is also possible that other fragmentation methods or lower-energy collisional activation could be employed to localize isomerization sites.

### Quantification of Isomerized Species in Mixture

**Figure 5:**
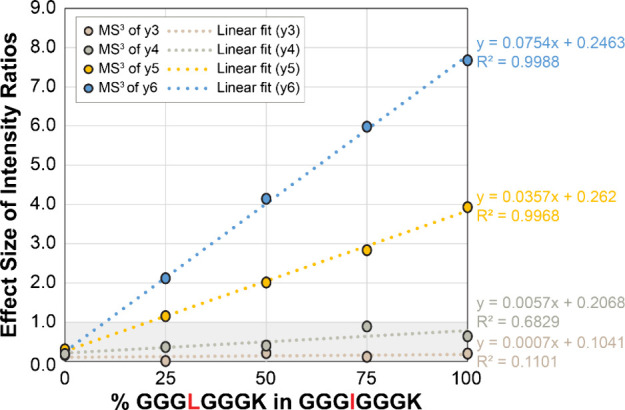
A mixture of different compositions of two peptide isomers, GGGIGGGK and GGGLGGGK, is compared to a pure GGGIGGGK standard. CID fragment ions containing isomerized residues y_5_^+^ (shown in yellow) and y_6_^+^ (shown in blue) were fragmented using CID in MS^3^. A highly correlated, linear relationship between the composition of isomers and the corresponding effect size was observed in both fragment ions. In contrast, CID fragments without the isomerized residue (y_3_^+^ and y_4_^+^) do not appear to be correlated and yield effect sizes below 1.0 for all compositions (gray shaded area).

We found the ability of collisional activation to distinguish Leu/Ile to be surprising given that fragmentation should be dictated by the mobile proton mechanism, and the two similar hydrophobic side chains would not be expected to influence proton location or mobility. To further confirm the results, we examined mixtures of the two peptides and calculated effect sizes relative to pure GGGIGGGK as shown in Figure 5. Differences in the effect size observed for both the y_5_ and y_6_ ions (which both include the relevant Leu/Ile) track as a function of the amount of each peptide that is present in the mixture. In fact, the correlation is linear, and such data could be used to quantify the amount of each isomer in a mixture without the need to separate isomers. These results are strong evidence that the side chains of Leu and Ile can influence fragment ion abundance following collisional activation even in the absence of other potentially interacting side chains.

Although our results demonstrate that isomer localization is frequently achieved in direct-infusion experiments, analysis of biological samples would typically involve the analysis after separation by liquid chromatography. Complete MS^3^ analysis of peptides in online experiments is unfeasible due to the number of peaks that need to be analyzed and the requirement for signal averaging. However, for peptides that have been mapped out in offline experiments, it is possible to program a targeted approach that can confirm the isomerization site with a few MS^3^ targets. To evaluate this concept, we acquired targeted data for FAEDVGSNK (l vs d-isoAsp) in an online experiment. MS^3^ data for 10 scans of the b_5_ and y_6_ ions yield effect size differences of 2.5 and 2.1, respectively (see Figure S3). As expected, these numbers are lower than the values obtained with greater averaging by direct infusion (b_5_ = 5.6, y_6_ = 5.3; see Figure 2), but these effect sizes are still sufficient for confident isomer localization. These results suggest that targeted online analysis is feasible for peptides where the fragmentation has been mapped out with direct infusion and the effect size differences are large and more likely to remain significant with reduced sample averaging.

## Conclusion

We demonstrated that mass spectrometry can be utilized to localize sites of isomerization in peptides. Although MS^2^ fragmentation can easily identify isomerized peptides by comparison to one another, MS^3^ fragmentation of the MS^2^ product ions can further localize the isomerized site to a smaller portion of the sequence or ideally a single residue. The method works best for peptides for which the MS^2^ spectra contain high-intensity fragments that also afford reasonable sequence coverage. In certain cases (especially for fragment ions composed of a few residues), the number of MS^3^ fragments is small, and differences in the overall spectra are unlikely to exceed statistical certainty. However, by comparison of the statistical reproducibility across many scans for the fragment ratios that differ most, certainty can be more readily ascribed to differences in MS^3^ spectra. Surprisingly, the method can localize isomeric species, such as Leu versus Ile, with functional groups that are not expected to directly affect the probative dissociation pathways. In other words, the hydrocarbon side chains of Leu/Ile are not expected to interact strongly with protons, yet they still perturb mobile-proton mediated fragmentation patterns sufficiently for detection. Importantly, this method enables localization of isomerized sites in peptides using only tandem-MS, which is widely available in many variations of commercial mass spectrometers. Furthermore, it may be possible to localize sites of isomerization in other classes of biomolecules with this same approach.

## Supporting Information Available

Additional experimental details, data, and spectra (PDF)
