# Supplementary material for: Localizing Isomerized Residue Sites in Peptides with Tandem Mass Spectrometry
Source: J Am Soc Mass Spectrom. 2024 Mar 5;35(4):705–13. doi: 10.1021/jasms.3c00373 (PMC10995990; doi:10.1021/jasms.3c00373)
Supplement: Supplementary file 1 — js3c00373_si_001.pdf [file js3c00373_si_001.pdf]

SUPPORTING INFORMATION

Localizing Isomerized Residue Sites in Peptides with Tandem Mass Spectrometry.

Hoi-Ting Wu, Brielle L. Van Orman, and Ryan R. Julian*

Department of Chemistry, University of California, Riverside, California 92521, United States

* Corresponding author: Ryan R. Julian

E-mail: ryan.julian@ucr.edu

Table of Content

Figure S1. The comparison of using different isomer differentiation methods.. ....S2

Figure S2. CID spectra of TLGPFYPSR peptide isomers. S3

Figure S3. LC-MS^3 experiment of a mixture of FAEDVGSNK All-L and D-isoAsp....................S4

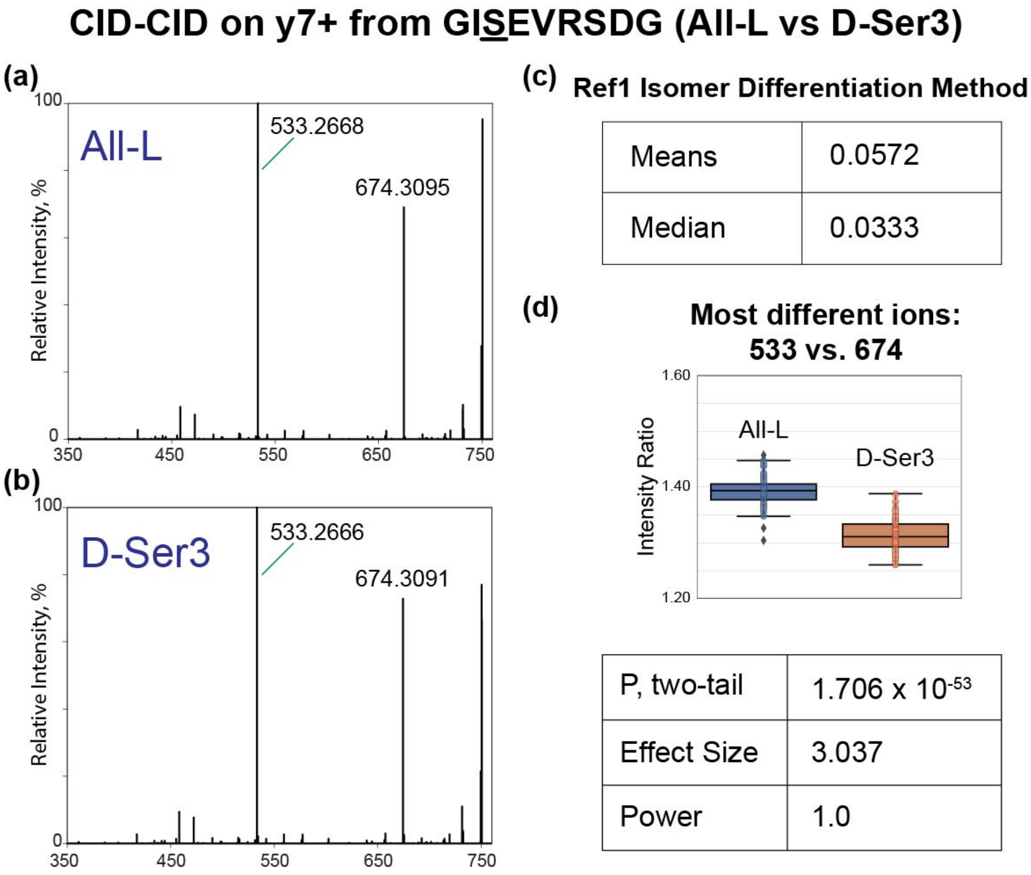

Figure S1. The comparison of using different isomer differentiation methods. MS^3 spectra from y7+ ion of GISEVRSDG peptide isomers **(a)** All-L and **(b)** D-Ser3 are shown. **(c)** Isomer differentiation method using Ref1[1]shows that there is no significant difference between the spectra. (d) Using the R-value ratio of 533 and 674 peaks identifies the spectra are indeed different between the isomers.

[1] Wu, H.-T.; Riggs, D. L.; Lyon, Y. A.; Julian, R. R. Statistical Framework for Identifying Differences in Similar Mass Spectra: Expanding Possibilities for Isomer Identification. *Anal. Chem.* **2023**, *95* (17), 6996–7005. DOI: 10.1021/acs.analchem.3c00495.

αA-crystallin [13]TLGPFYPSR[21]

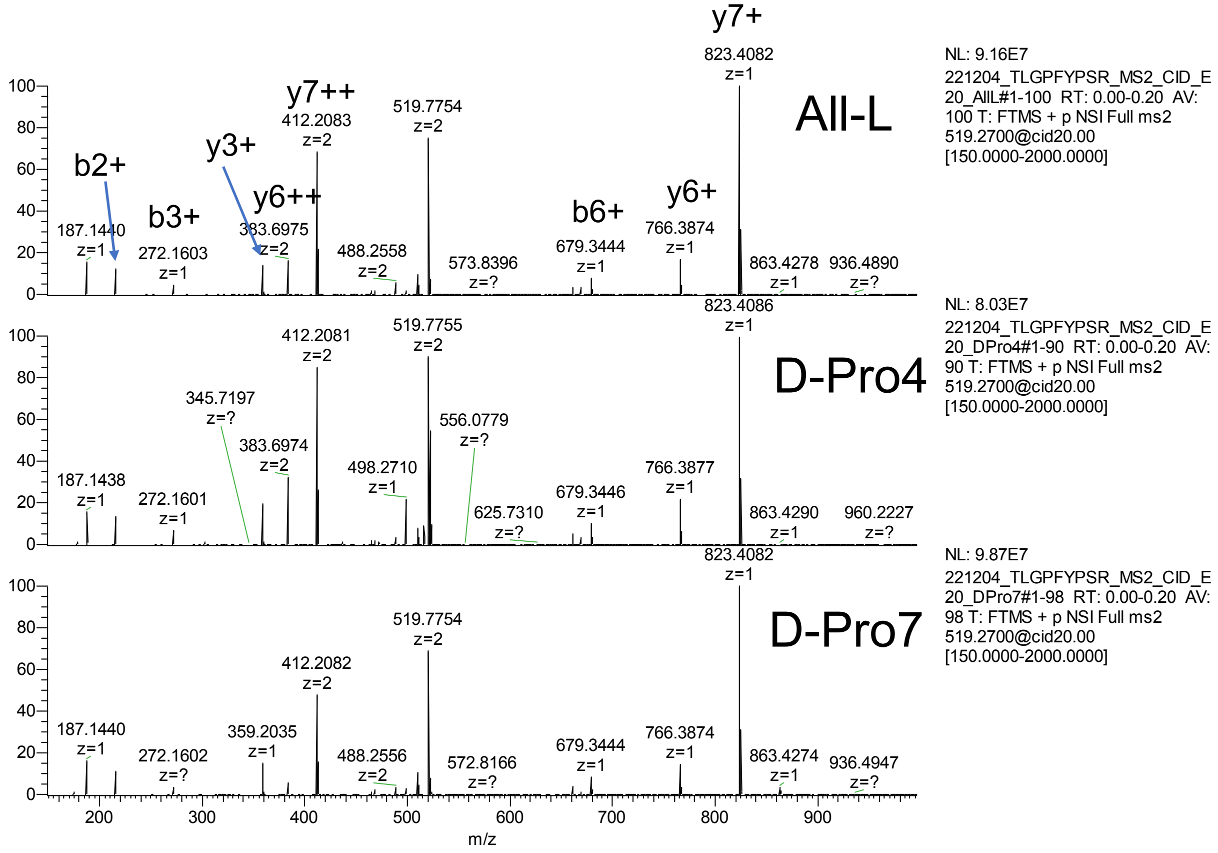

Figure S2. CID spectra of TLGPFYPSR peptide isomers.

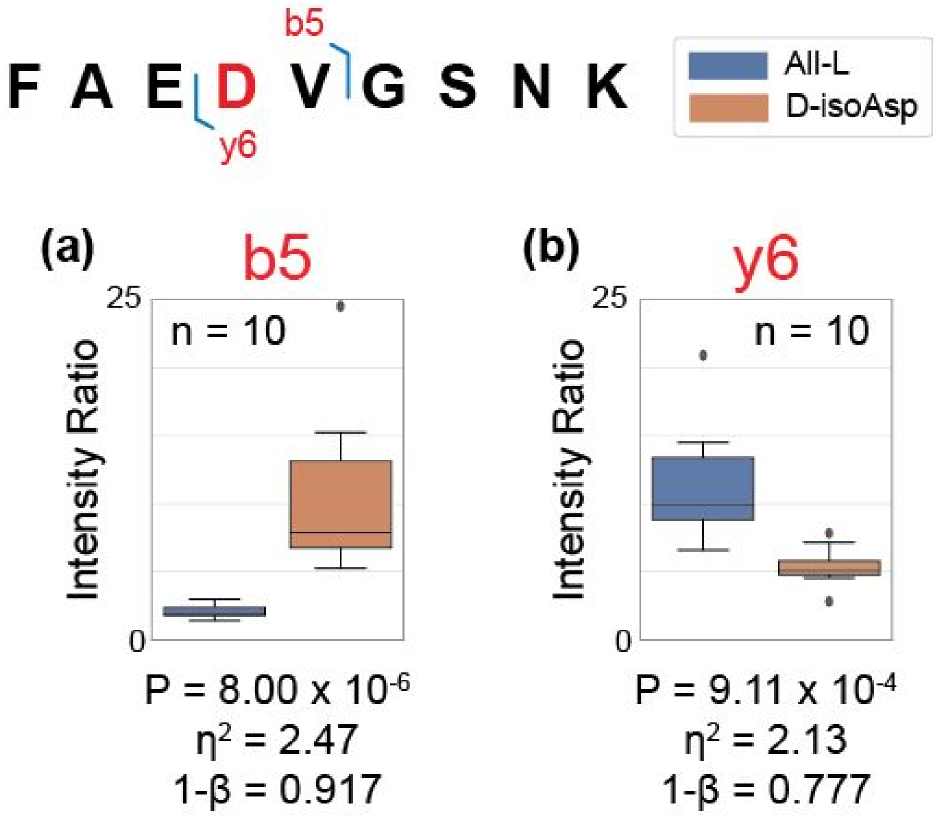

Figure S3. LC-MS[3] experiment of a mixture of FAEDVGSNK All-L and D-isoAsp. Fragments containing isomerized Asp (b_5^+ and y_6^+) have effect sizes of a) 2.47 and b) 2.13, suggesting the isomerized residue's locations to be $^4DV^5$.
